# Coral growth, retraction, defense, and regenerative strategies revealed by live microCT

**DOI:** 10.1126/sciadv.aee3183

**Published:** 2026-05-27

**Authors:** Karina Araslanova, Marketa Kaiser, Anton Fetisov, Diana Gavrik, Tomas Zikmund, Daniel Abed-Navandi, Karel Katovský, Jozef Kaiser, Igor Adameyko

**Affiliations:** ^1^ Department of Neuroimmunology, Center for Brain Research, Medical University Vienna, 1090 Vienna, Austria.; ^2^ Central European Institute of Technology, Brno University of Technology, 601 77 Brno, Czech Republic.; ^3^ Department of Functional and Evolutionary Ecology, Faculty of Life Sciences, University of Vienna, 1030 Vienna, Austria.; ^4^ Haus des Meeres, 1060 Vienna, Austria.; ^5^ Faculty of Electrical Engineering and Communication, Brno University of Technology, 61200 Brno, Czech Republic.; ^6^ Department of Physiology and Pharmacology, Karolinska Institutet, 17177 Stockholm, Sweden.

## Abstract

Understanding how corals build and remodel their skeletons is key to explaining reef resilience, yet most insights come from static imaging. Using longitudinal live microCT, we tracked the same coral colonies over weeks to months at micrometer resolution. Coral skeleton formation is not a uniform accretion process but a dynamic integration of multiple programs, including vertical and horizontal patterned growth, previously undescribed defensive wall-building against competitors, exploratory edge behavior with reversible expansions and retractions, and skeletal regeneration favoring rapid, imprecise yet effective matrix expansion. Time-resolved imaging links colony-scale growth to microscale events, showing that all modes depend on balances between fusion of skeletal microparticles and layered matrix deposition, guided by tissue prepatterning. Beyond corals, this framework generalizes to studying skeletal dynamics across diverse biomineralizing organisms.

## Teaser

Live microCT approach reveals how corals construct defensive walls, explore surroundings with reversible growth, and regenerate.

## INTRODUCTION

Coral skeletons form the three-dimensional (3D) framework of reef ecosystems, storing ecological history and supporting immense biodiversity. Yet, despite their ecological and geochemical importance, the spatiotemporal dynamics by which corals build, remodel, and repair their skeletons remain incompletely resolved (*1*, *2*). A full understanding requires integrating observations across multiple scales, from the macroscopic architecture of the colony down to nanoscale crystallization events.

Historically, many assumptions about coral skeletal growth and environmental response have been based on static, end-point methods (*3*–*6*). Thanks to the fact that the coral skeleton inherently records its own formation history (*7*–*9*), dynamic processes can be reconstructed by analyzing the structure of end-point samples using electron microscopy, nanoscale secondary ion mass spectrometry (NanoSIMS), microcomputed tomography (microCT), spectroscopy, or related techniques (*6*, *10*–*18*).

Collectively, these methods, along with biomineralization studies, have produced a model of coral skeleton growth (*19*). In this model, the calicoblastic epithelium transports amorphous calcium carbonate (ACC) to the extracellular calcifying medium and secretes skeletal organic matrix (SOM) (*20*–*26*). SOM consists of polysaccharides, lipids, and proteins, which scaffold ACC crystallization (*27*–*29*). Von Euw *et al.* (*30*) mapped the distribution of organic molecules and inorganic phases of the coral skeleton using synchrotron-based spectromicroscopy, showing that ACC is stabilized by SOM-proteins in specific zones [i.e., early mineralization zones (EMZs)/centers of calcification (CoCs)] before transforming to aragonite. Great efforts, including genomic, transcriptomic, and proteomic analyses, were made to investigate the composition of SOM and to analyze the “calcification toolkit” across different species (*31*–*33*). During primary skeletogenesis, this process leads to the formation of spherulite-like structures that subsequently fuse together, creating primary septa and the basal plate (*33*). In adult colonies, spatial and temporal inconsistencies of this process across the skeleton result in a biphasic architecture, described as the “two-step” growth model (*30*, *34*).

The first component of this model consists of EMZs containing CoCs, where calcification is initiated, and elongated aragonite fibers emerge, grow, and connect neighboring CoCs (*25*, *35*, *36*). These regions are usually visible in sections of skeletons, where they form lines in septa called rapid accretion deposits/fronts (RADs/RAFs), fast-calcifying regions found in rapidly growing structures of corallites (*37*, *38*). The second component comprises thickening deposits (TDs), which are slower-growing, flatter zones of layered fibrous aragonite located between corallites. TDs and RADs also differ in chemical composition. Fibrous zones (in TDs) are characterized by a lower organic sulfate content compared with CoCs (in RADs) (*39*, *40*).

When it comes to the methodology for such exploration, crystal formation dynamics can be resolved with NanoSIMS, offering great opportunities for studying biomineralization on the nanoscale level (*4*, *41*). Sclerochronology, 3D surface imaging and dye-pulse labeling coupled with computational modeling were used to reconstruct and describe colony-wide growth patterns (*4*, *10*, *11*, *42*–*47*). Although the outcome of adult skeletal growth is well described, the real-time dynamics of RAD and TD formation remain ambiguous due to the need for longitudinal, live-imaging studies.

Additionally, the mesoscale processes by which corals add polyps to form colonies, expand across substrates, compete, regenerate, grow vertically, and develop species-specific branching architectures remain inferred rather than directly observed. A central challenge is capturing oscillatory and transient events in the same individual over time. Cycles of skeletal building via irreversible remodeling, as well as timely responses to predators, pathogens, competitors, or environmental change, including adaptation, tissue regeneration, and morphological adjustment, cannot be reconstructed from end-point analyses alone.

To address these issues, live imaging with optical microscopy or stage-by-stage scanning electron microscopy (SEM) imaging has been performed on newly settled primary polyps and enabled studies of initial skeleton formation, allowing researchers to capture biomineralization dynamics and to investigate how these dynamics change under different environmental factors (*6*, *48*, *49*). The combination of advanced approaches, like coral-on-a-chip, with light microscopy enabled dynamic studies of calcification in primary polyps in a controlled environment (*50*).

Nevertheless, these previously existing live approaches are not feasible for larger, nontransparent, 3D colonies, where multiple polyps integrate growth and where the whole colony transiently responds to external stimuli. This gap is particularly critical for larger and older corals with complex, 3D skeletons, whose diverse growth patterns have never been captured at great resolution in vivo in real time.

The increasing toolkit of microCT approaches offers endless possibilities for biomineralization research and for uncovering the responses of this process to different environmental parameters, like acidification (*51*). However, all existing CT-based approaches are static and miss these temporally variable behaviors because each measurement is destructive or derived from a different specimen. Therefore, to resolve fundamental questions about coral growth and biomineralization, CT-based methodologies enabling continuous, direct observation of living skeletal formation at the micro- and mesoscales over days to months are needed. Until now, the prevailing assumption that repeated x-ray exposure would be lethal to coral tissues and symbionts (an extrapolation from vertebrate models) has prevented the application of longitudinal live microCT to mature corals.

Here, we challenge this assumption by leveraging advances in microCT instrumentation and low-dose protocols. Our findings demonstrate that corals and their symbionts can tolerate repeated imaging without most detrimental consequences. This methodological shift opens the door to dynamic, nondestructive studies of coral growth, repair, and morphogenesis at advanced developmental stages and under diverse environmental conditions. Specifically, our longitudinal live microCT approach enables repeated imaging of the same coral colony with micrometer-scale resolution, making it possible to visualize skeletal fusion and accretion processes as they unfold in real time. By combining repeated photography, time-resolved CT imaging of coral skeletons, and chemical contrasting of soft coral tissue, we reveal distinct skeletal programs mediating defensive strategies and repair in response to competing organisms, as well as explain how corals explore the environment by directional and oscillating growth. Using time-resolved 3D reconstructions, we further establish the logic for de novo polyp initiation and formation of species-specific 3D morphologies. In sum, the dynamic development of coral colonies over time, their challenges, and responses via oriented skeletal growth become traceable with live microCT approach, especially in combination with other methods modeling ecological contexts.

## RESULTS

### Live microCT imaging enables longitudinal studies of coral growth

To investigate the logic and modalities of coral skeletal expansion, we focused on two reef-building coral species: *Stylophora pistillata* and *Pocillopora damicornis*. These species were selected because they combine experimental tractability with biological diversity: Both are readily maintained in aquaculture and phylogenetically related, facilitating generalization of shared principles. Moreover, *S. pistillata* is emerging as a popular scleractinian model, with a fully annotated genome and single-cell transcriptomic atlases of cell types, which provides a molecular framework for interpreting observed structural dynamics (*52*).

**Fig. 1.**
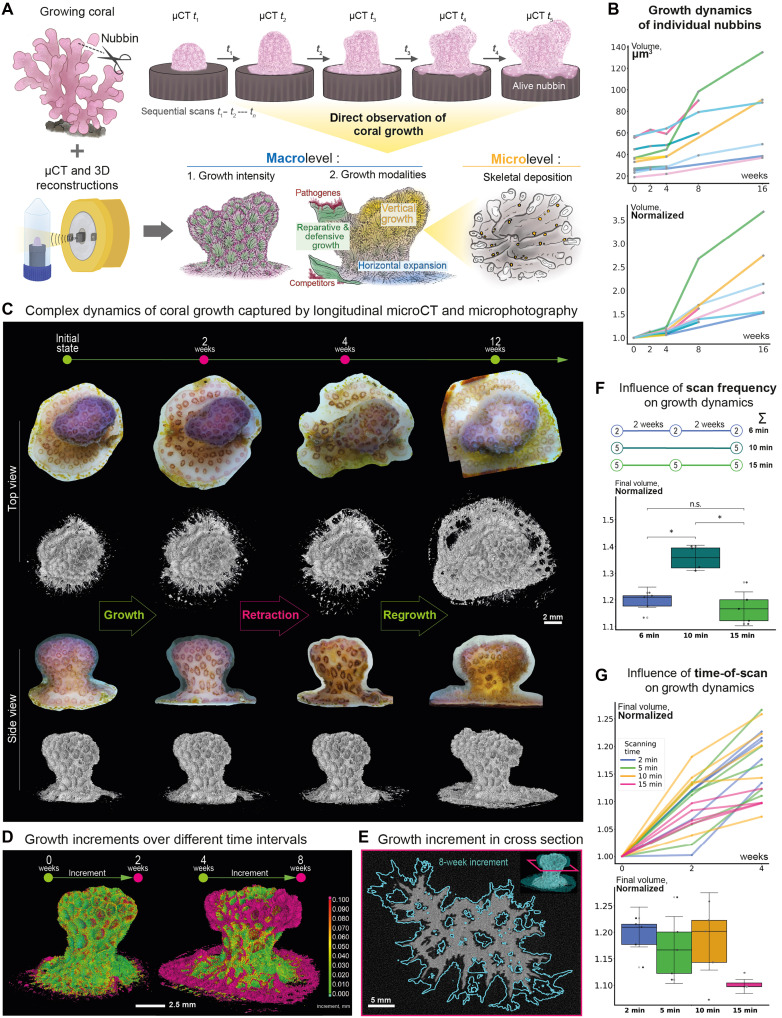
Establishment and validation of a longitudinal live microCT approach for visualizing skeletal growth of the same growing coral in 4D. (**A**) Schematic illustration of the nondestructive, repetitive scanning of coral nubbins growing over time. (**B**) Absolute (top) and normalized (bottom) skeletal volume growth trends of eight individual nubbins over 16 weeks. (**C**) Longitudinal live microCT captures the complex growth dynamics of coral colony in the timescale of weeks and months. Sequential 3D reconstructions reveal distinct phases of initial expansion, skeletal retraction, and subsequent regrowth. Scale bar, 2 mm. (**D**) Colored 3D maps depict the volume of new skeleton deposited during shorter (0 to 2 weeks) and longer (4 to 8 weeks) time intervals. Colorbar represents the magnitude of each growth increment in millimeters. Scale bar, 2.5 mm. (**E**) Cross section through two aligned 3D reconstructions demonstrating increment of skeletal growth. Red plane depicts the position of the cross section on the 3D models. (**F**) Schematic timeline of three microCT scanning regimes varying by scan duration, frequency of scans, and total scan duration (top). Final normalized skeletal volume of coral nubbins for each regime (bottom). Data are presented as means ± SD. The regime with less frequent scans resulted in final volume significantly different from two other regimes (two-sided Mann-Whitney-Wilcoxon test with Bonferroni correction, *P = 4.762 × 10^−2^). n.s., not significant. (**G**) Volume represented by growth trajectories (top) and final normalized skeletal volume (bottom) of coral nubbins scanned under four regimes with different scan duration. Data are presented as means ± SD.

During the study, we established and optimized live microCT scanning protocols that minimized both physiological stress and radiation exposure. At the Haus des Meeres aquarium (Vienna), we implemented a system of coral fragmentation and rearing based on our previously described approach (*53*–*55*). Briefly, small coral fragments (7-mm “nubbins”) derived from larger colonies were affixed to plastic holders and maintained under controlled aquarium conditions until they reached sufficient stability for repeated imaging. Colonies expanded successfully and tolerated multiple rounds of scanning, allowing longitudinal datasets of skeletal growth (Fig. 1, A to E). Corals showed horizontal and vertical growth with dynamic properties such as oscillating retraction and expansion of soft tissue and skeletal matrix (Fig. 1C).

Optimization of imaging parameters was critical. The number of scans per month directly influenced subsequent growth rates in both investigated species (Fig. 1F and fig. S1), while scan duration and x-ray dose (Fig. 1G) also affected colony expansion. Based on these results, we identified an optimal scanning regime that balanced temporal resolution with coral health, enabling repeated volumetric reconstructions over weeks and months without compromising survival (fig. S1 and movie S1).

Despite these advances, some limitations remained. The achievable temporal resolution is inherently constrained by scan duration and required recovery intervals (1 week to 2 months) (Fig. 1, D to E); secretion events occurring on timescales shorter than our imaging frequency could therefore be undersampled. Furthermore, while microCT resolves deposited mineral structures, it does not directly capture the cellular or molecular processes underlying deposition. Nevertheless, the establishment of minimally invasive protocols for longitudinal imaging represents a major advance, enabling direct visualization of skeletal growth dynamics in living corals.

### Coral colonies activate a defensive skeletal program when responding to competitors

**Fig. 2.**
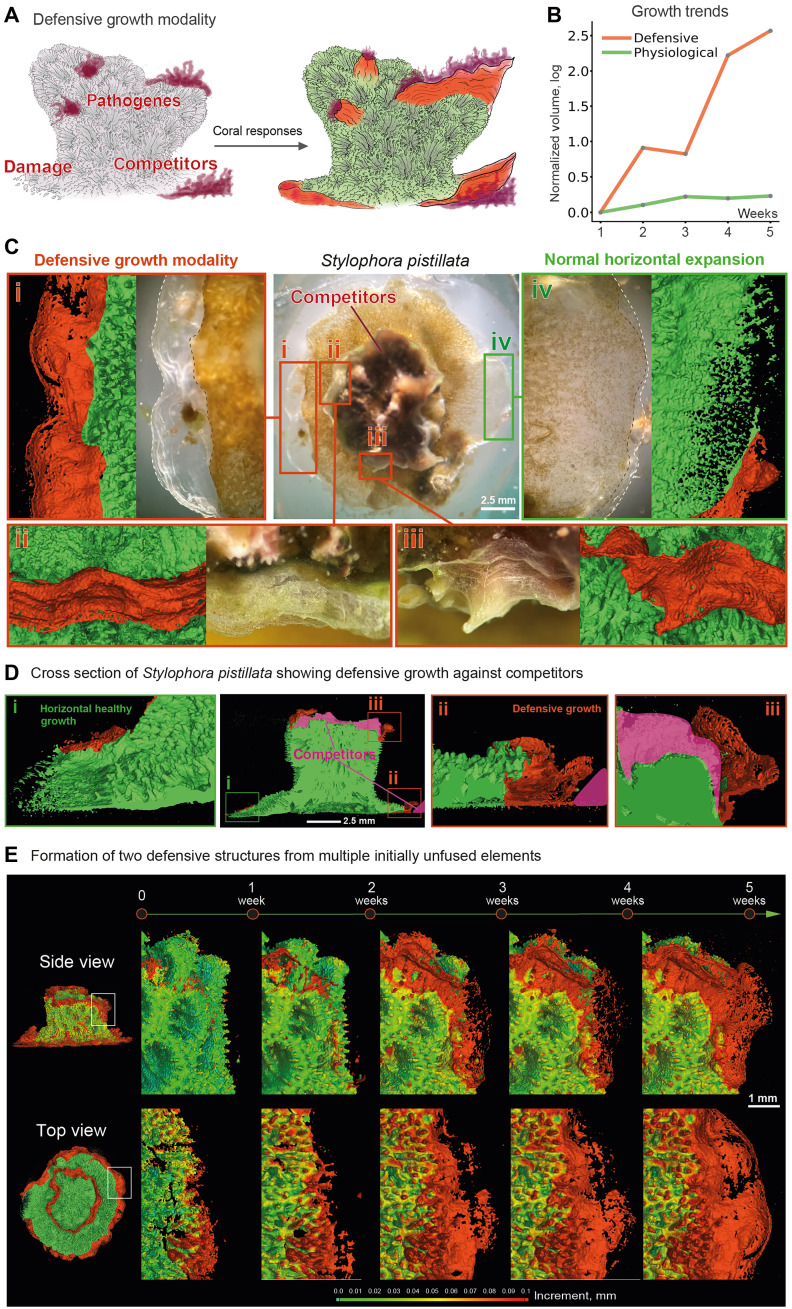
Corals build skeletal walls to defend from expansion of competitors. (**A**) Schematic illustration of defensive growth representing structures built by a colony in response to invading or competing organisms. (**B**) Comparison between speed of growth in physiological and defensive modality shown by relative volume increase (normalized by volume at second measurement and log scaled). Defensive structures were manually segmented from skeletons. (**C**) Defensive growth occurs in places of contact with competitors and characterized by (i) flat continuous skeletal layers and lack of symbionts in soft coral tissue [compare with physiological nondefensive growth in (iv) showing patterned matrix allocation and covered by living tissue with symbionts]. (ii and iii) Defensive growth in response to algae invading skeleton on top of the nubbin. (**D**) (i) Normal physiological horizontal growth has a rugged pattern. Microparticles are allocated on top of substrate and expand strictly in 2D plane. (ii) Defensive growth at the colony border on substrate surface is characterized by flat, nonpatterned structure of skeletal matrix and margins elevated above substrate. (iii) Defensive skeletal walls on top of the nubbin extending outward to isolate the compromised area. (**E**) Time-resolved dynamics of defensive growth shows the stages of defensive structures formation: nonpatterned allocation of initially unconnected microparticles, their fusion, closure of holes, and formation of flat and continuous wall.

After optimizing scanning conditions, coral nubbins were imaged at different intervals to examine their growth dynamics. To exploit the longitudinal nature of this approach and gain insights into temporally controlled processes not accessible by end-point methods, we exposed *S. pistillata* colonies to suboptimal conditions that included colonization by turf diatoms (*56*). As diatoms began invading and killing patches of live tissue, the coral mounted a systemic and fast defensive response: It erected translucent skeletal walls that physically separated compromised regions from the rest of the colony (Fig. 2, A and B, and figs. S2 and S3). Longitudinal microCT revealed that these walls were not formed by simple layered accretion of skeletal matrix. Instead, a thin layer of overlying tissue initiated the deposition of unfused microparticles of varying sizes, which subsequently fused into a continuous barrier (Fig. 2, C to E). This stepwise assembly unfolded over several weeks and successfully isolated the diatom-colonized zones (Fig. 2D). We define these defensive structures by flat and continuous nature (Fig. 2C, i to iii); lack of polyps, gastrovascular canals, and photosynthetic symbionts (compare Fig. 2C, i and iv); and lack of microarchitectural features (spikes and ridges) (compare Fig. 2D, i and ii and iii), suggesting that the defensive growth operates outside the normal trophic architecture of the colony. Notably, the protective walls expand more rapidly than other regions, indicating a colony-wide redistribution of resources in response to danger (Fig. 2B). Over time, some of these walls can be either conquered by competitors (fig. S2) or remodeled into typical living tissue, gradually acquiring symbionts and nascent polyps. The transient exclusion of symbionts from protective structures may reflect their sensitivity to toxins or other harmful metabolites produced by invading organisms. It also points to a potential trade-off between immediate defense and energy acquisition through symbiosis. Together, these observations demonstrate how ecological stressors feed back into coral skeletogenesis, triggering specialized defensive programs of matrix deposition and remodeling that can later reintegrate into regular colony growth.

### Coral colonies expand through horizontal and exploratory growth

**Fig. 3.**
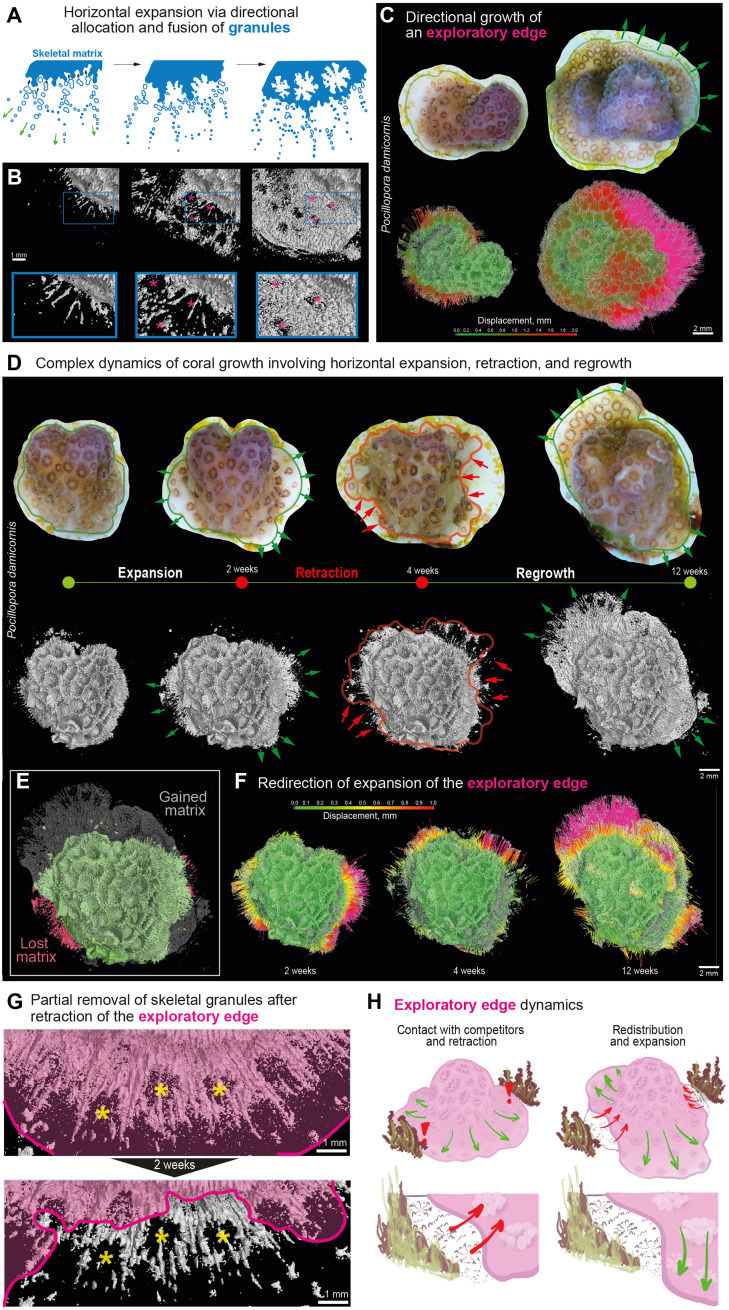
Longitudinal microCT reveals dynamic nature of coral horizontal growth. (**A**) Schematic illustration conceptualizing horizontal growth modality of coral colonies. (**B**) Expansion of colony margin in horizontal plane shown by sequential microCT close-up views. Pink asterisks indicate positions of individual polyps forming in soft tissue. Scale bar, 1 mm. (**C**) Displacement map of the advancing growth margin over time, derived from aligned sequential microCT scans. The colorbar indicates the magnitude of hard tissue advancement (in millimeters), revealing that 2D expansion is not uniform but advancing in one favorable direction (green arrows). Scale bar, 2 mm. (**D**) Sequential top views from longitudinal microCT imaging over 12 weeks show a cycle of horizontal expansion (0 to 2 weeks, green arrows), retraction (2 to 4 weeks, red arrows), and regrowth (4 to 12 weeks, green arrows). Scale bar, 2 mm. (**E**) Overlay of microCT 3D models from the initial (week 0) and final (week 12) time points highlights areas where skeletal matrix was gained (gray) and lost (pink) over the entire retraction-regrowth cycle (12-week period). (**F**) Sequential displacement maps demonstrating dynamical redirection of colony horizontal expansion at the exploratory edge over time. The colorbar indicates the magnitude of hard tissue advancement (in mm). Scale bar, 2 mm. (**G**) MicroCT close-up view of the growth margin after a 2-week period showing the loss of previously deposited skeletal microparticles after retraction of living tissue (pink). Positions of polyps are indicated by yellow asterisks. Scale bar, 1 mm. (**H**) Schematic illustration representing the exploratory edge concept.

The construction of defensive walls contrasts with the regular horizontal expansion observed in all growing nubbins of *S. pistillata* and *P. damicornis* (Fig. 3, A and B, and fig. S4). Consecutive weekly, biweekly, and monthly scans, combined with live photography, showed that the healthy advancing growth margin rapidly becomes inhabited by photosynthetic symbionts, while skeletal deposition proceeds in a patterned way, which creates grooves and articulated skeletal elements. However, similar to the defensive growth, horizontal expansion occurs through the placement of discrete, unconnected skeletal microparticles, conceptually similar to previously reported spherulites (*16*, *57*) and microdumbbells (*6*), that gradually fuse to form a continuous framework across the expanding margin (Fig. 3B).

Correlative imaging (microCT and microphotography) demonstrated that polyps are induced and initiated de novo into a juvenile stage while the skeletal matrix is still very sparse and is not specifically patterned for positions of polyps. Thus, if assessed solely by skeletal structure, early polyp positioning would remain invisible (Fig. 3B, polyp positions marked by asterisks). As deposition progresses and microdumbbells fuse, corallites and polyp positions become clearly defined, showing that skeletal morphogenesis follows cues set by soft tissue expansion and polyp induction (Fig. 3B and fig. S4). Both species exhibited similar horizontal expansion dynamics, suggesting a conserved program in which corals prepattern soft tissue architecture and then consolidate it through calcified matrix deposition (Fig. 3C and fig. S4).

Next, our longitudinal approach uncovered dynamic growth capabilities of newly establishing coral colonies. When settling, new colonies rapidly expand horizontally and conquer free space in 2D (Fig. 3C). When faced with unfavorable local conditions and the presence of competing organisms, corals induce retraction of the living tissue front at the colony edge (Fig. 3D). Tissue withdrawal is accompanied by loss of hard skeletal matrix in such areas: Portions of the previously deposited skeleton were likely resorbed and their mineral content redistributed across the colony (Fig. 3, E to G). After retraction, corals expand in directions free from competitors and redirect their growth toward more favorable areas (Fig. 3F). Thus, the dynamics of growth at expanding margins of coral colonies can be described as exploratory behavior. Rather than proceeding as a uniform wave of expansion, the colony margin displayed pulses of advance and retreat, as if “probing” the environment and even committing to partly reversible skeletal deposition (Fig. 3H). This dynamic resembles the exploratory logic of axonal growth cones in developing nervous systems, which extend and retract filopodia to navigate toward synaptic targets, or the navigation of slime molds, which optimize migration paths across complex terrains by dynamically balancing extension and retraction (*58*, *59*). In this 2D growth, tissue expansion and skeletal deposition are selectively channeled into advantageous zones while avoiding energetically costly or ecologically hostile territories. This process does not involve stacking of polyp chambers while operating in 2D plane and therefore does not contribute directly to 3D morphogenesis or the species-specific shaping of the colony.

### Coral colonies switch to 3D morphogenesis via vertical growth

**Fig. 4.**
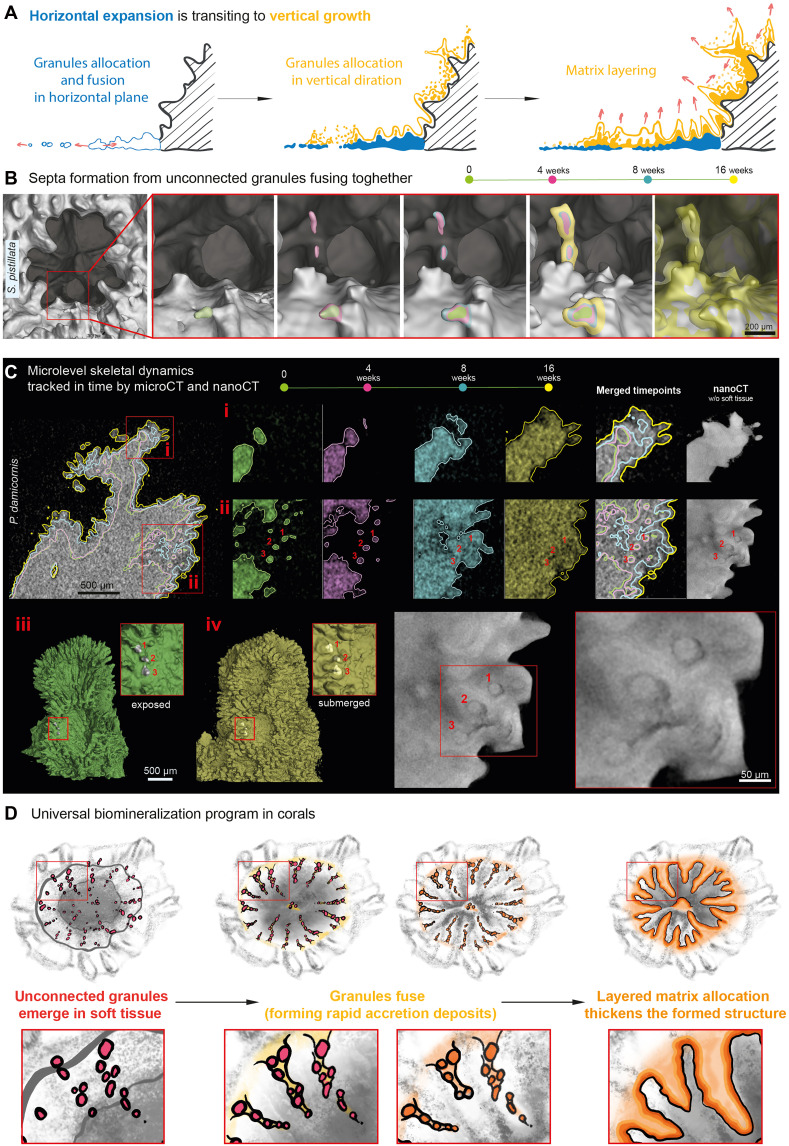
NanoCT and longitudinal microCT reveal the universal mechanism of vertical growth in corals. (**A**) Schematic illustration of transition from horizontal growth to vertical growth. (**B**) Process of septa formation captured over time includes granule formation, fusion, and further thickening. Color masks encode the sequential scans made over 16 weeks. Scale bar, 200 μm. (**C**) MicroCT reconstructions show sites of granule fusion (i) and spikes growth (ii). High-resolution nanoCT scan reveals the internal traces and morphology of the same skeletal regions (*1*, *2*, *3*) that were initially exposed (iii) but became submerged (iv) within the consolidated skeleton in the course vertical growth. (**D**) Schematic illustration conceptualizing the biomineralization program in corals.

In contrast, vertical growth is defined by skeletal extension above the horizontal plane and driving the 3Darchitecture of the colony (Fig. 4A). Our longitudinal datasets revealed that vertical expansion begins with the appearance of unconnected skeletal particles proximal to the main surface (Fig. 4B). Their size overlaps with the particles observed in horizontal expansion, and the irregularities of early corallite surfaces corresponded closely to particle dimensions, indicating that these features arise from particle fusion events. The longitudinal design of the study allowed us to directly observe such fusion across consecutive scans, for example, in the context of forming septa (Fig. 4B), providing strong support for this mechanism. Unlike horizontal expansion, however, vertical growth incorporates an additional step, in a vertical dimension. After initial particle fusion, the surfaces are further smoothed, vertically expanded, and consolidated through a layered matrix accretion, consistent with the two-step model of coral growth (*30*, *34*). This layered matrix allocation follows the original pattern set up by the fused particles in both horizontal and vertical direction, in agreement with RAD-TD terminology (Fig. 4C) (*37*, *38*, *40*). Here, we propose that these zones of initial particle attachment represent rapid accretion deposit regions, where the matrix allocates faster and forms “spikes” and “ridges” patterned in a species-specific vertical manner (Fig. 4D).

### Live microCT reveals how polyp initiation defines colony architecture

**Fig. 5.**
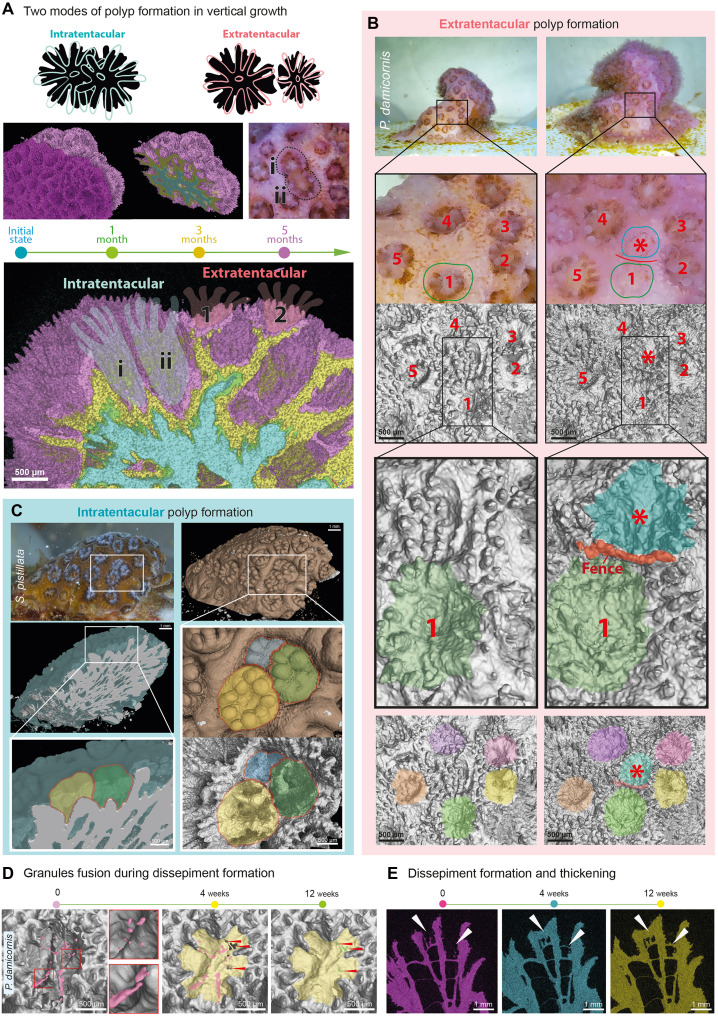
Live microCT allows tracking two distinct modes of new polyp formation that contribute to the 3D colony structure. (**A**) A cross section through four aligned sequential microCT scans and schematic diagrams illustrating extratentacular (new polyp forms new cup; 1 and 2) and intratentacular (two polyps share one cup; i and ii) types of polyp formation. Scale bar, 0.5 mm. (**B**) Extratentacular polyp formation in *P. damicornis*, visualized through two sequential microCT scans with a time interval of 2 months. The scans show de novo polyp initiation (marked by red asterisk) and the “fencing” of its cup (labels 1 to 5 indicate same polyps in two time points). (**C**) Intratentacular polyp formation in *S. pistillata*, revealed by aligned microCT scans of the skeleton and phospho-tungstic acid (PTA)–stained soft tissue, capturing two new polyps that share a cup with the mother polyp. (**D**) Formation and further thickening of horizontal dissepiment forming “new floor” of the old growing polyp cup over 12 weeks. Initially unconnected microparticles (pink) lying in one 2D plane fuse into solid and thin skeletal membrane (yellow). (**E**) Dissepiment thickening is evident in optical cross sections. Arrowheads point to the forming and thickening dissepiment.

Furthermore, at the macroscale, vertical growth is driven by the repeated initiation and stacking of new polyps above existing ones, generating an inherently 3D and anisotropic process. Longitudinal imaging that combined microCT with surface photography revealed a consistent temporal sequence: Polyps first appeared within the soft tissue, identifiable by tentacles and basic anatomy, before any skeletal signatures became detectable in 3D reconstructions. This pattern held true for both extra- and intratentacular modes of polyp formation (Fig. 5A). In extratentacular formation, new polyps emerged de novo on flat tissue patches spanning between older polyps [Fig. 5, A (polyps 1 and 2) and B]. During our longitudinal analysis of coral growth, such patches tended to increase their surface area gradually, making space for initiation of new polyps. This is reminiscent of the situation with the horizontal growth, where the new polyps are induced at specific distances from each other (*60*). Also, similar to horizontal growth, only after polyp anatomy became evident in photographs did the skeleton begin to remodel the surrounding surface, first by raising subtle edge elevations and producing high numbers of unconnected and connected microskeletal elements and then progressively deepening a cup-shaped depression into a more mature and recognizable chamber (Fig. 5B).

Intratentacular polyp formation, by contrast, involved budding resulting in two or more polyps initially sharing a single skeletal chamber before partitioning into separate compartments [Fig. 5, A (i and ii) and C]. In addition to the longitudinal analysis based on the surface photography and microCT skeletal reconstructions, we also took advantage of the soft tissue contrasting with phospho-tungstic acid (PTA) to recover the anatomy of every individual polyp in relation to the 3D skeletal arrangement. This method helped to identify situations where two or three polyps were sharing one large chamber while generating nascent individual skeletal separations (Fig. 5C).

Last, once polyp chambers become too deep during growth, the polyps must lift themselves and form a dissepiment functioning as a new floor for the lower part of the polyp (Fig. 5, D and E). The observed formation of an endothecal dissepiment (*61*), a dissepiment forming within polyp corallites, appeared similar to defensive and horizontal growth, being a sequence of fusion events connecting the individual skeletal elements initiated within the horizontal layer of soft tissue (Fig. 5, D and E). These results are consistent with previous findings, where NanoSIMS was used to show that dissepiment formation results from rapid RAD formation, which is then followed by accretion to produce a complete mineralized plate (*62*). In our observations (Fig. 5, D and E), emerging and fusing particles form structures closely resembling RADs, which constitute the basal part of the dissepiment and are quickly expanded according to the logic of extending TDs.

In all cases, the polyp initiation was spatially localized rather than random, occurring at discrete “hotspots” on the colony surface. The recurrent activation of such sites produced a pulsed, anisotropic pattern of growth that likely underpins species-specific colony architectures. Branching, plating, or bushy morphologies may thus emerge from the frequency, spatial distribution, and reuse of initiation sites, together with skeletal sculpting guided by tissue-level cues (*45*). In this way, the macroscale anisotropy of coral colonies reflects the coordinated interplay of polyp initiation and subsequent skeletal remodeling.

### Live microCT shows the sequence of regeneration and repair of coral colonies

**Fig. 6.**
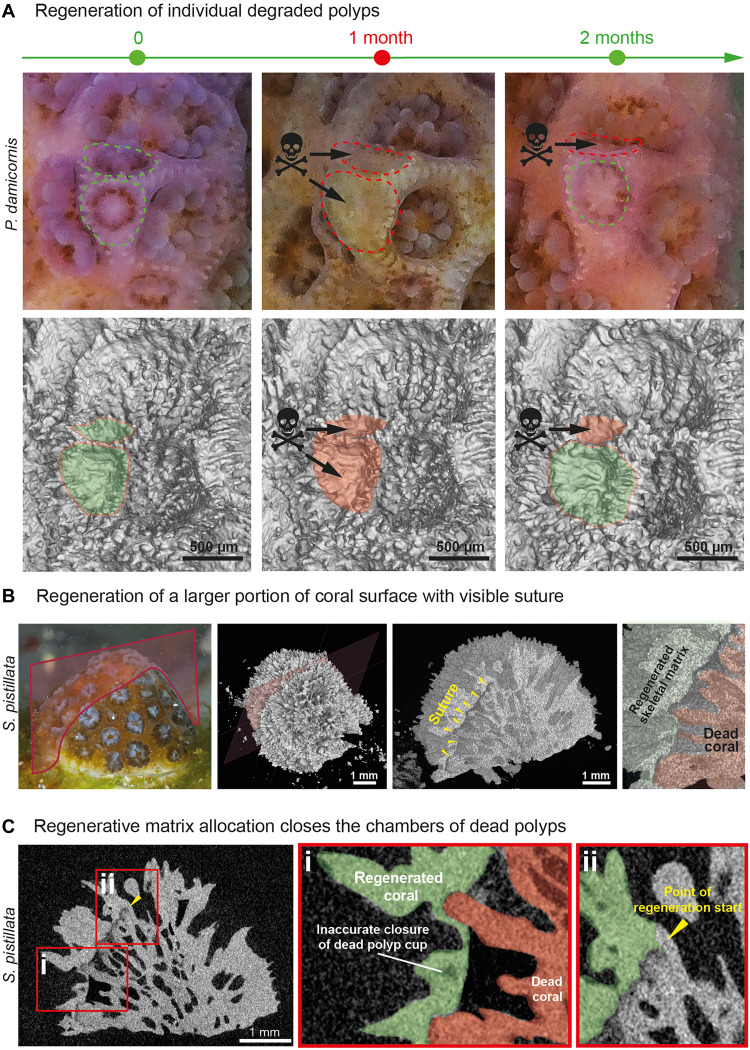
Coral colonies revealed two distinct regenerative strategies depending on the size of damaged area. (**A**) Sequential microCT scans and photos of *P. damicornis* nubbin over 2 months captured localized degradation of two polyps and corallite growth cessation (1-month time point), followed by the regeneration of a new polyp in the preexisting cup and the restoration of the cup growth (2-month time point). Scale bars, 500 μm. (**B**) MicroCT scans and photos of *S. pistillata* nubbin showing the regeneration of a large portion of the coral surface (in green), where extensive polyp death happened (in red), leaving a visible suture line between the dead area, and the newly regenerated tissue (yellow arrowheads). (**C**) Magnified optical cross section through the regenerated area. Note that the regenerated skeleton does not replicate the fine architectural pattern of the dead corallites (i and ii).

When local damage occurred within the 3D skeletal framework, coral nubbins displayed a robust regenerative capacity, often leaving no visible traces once the tissue had healed. When the overlying tissue remains intact but individual polyps resorb, new polyps often reemerge at the same site, reusing the preexisting skeletal morphology, including the original cup structures. In these cases, little to no remodeling of the skeletal surface is observed (Fig. 6A). On the other hand, when patches of live tissue encompassing few polyps died, the colony initiated a rapid repair program characterized by overgrowth of the damaged area and closure of the empty skeletal cups (Fig. 6B). The regenerating colony did not attempt to replicate the fine architectural details of the dead skeletal structure; instead, it acted pragmatically, sealing off the irregular substrate and establishing a uniform foundation for new polyp formation (Fig. 6C). The newly formed polyps subsequently occupied shallower skeletal cups compared with their older neighbors, indicating a priority for speed of recovery and functional restoration over precise morphological fidelity. This strategy underscores the colony’s bias toward reestablishing viable tissue coverage and symbiont-bearing polyps as quickly as possible, rather than reusing and reconstructing previously created skeletal architecture.

## DISCUSSION

Our longitudinal microCT framework reveals previously inaccessible dimensions of coral skeletogenesis, exposing dynamic and oscillating context-dependent programs of skeletal deposition that operate across time and spatial scales. Repetitive, nondestructive 3D imaging of the same colonies over time showed that coral growth is not a single, continuous process but rather a repertoire of coordinated modalities—defensive deposition, lateral expansion and retraction of the “searching colony edge,” vertical growth, new polyp morphogenesis, and regenerative growth over dead parts of the coral, all achieved via stitching of discrete microparticles [conceptually similar to spherulites (*16*, *57*) and microdumbbells (*6*)] in combination with layered accretion.

The observation that defensive walls are constructed from unfused microparticles that later adjoin to form continuous structure suggests a modular assembly logic rather than homogeneous accretion. Such a strategy is consistent with localized, tissue-mediated deposition by the calicoblastic epithelium and with hypotheses invoking particulate or irregularly-shaped precursors packaged and released in pulses (*63*, *64*). Defensive growth may be particularly critical during early stages of larval settlement or colony propagation via fragmentation, when small coral fragments are frequently falling into suboptimal environments (*65*, *66*). In such shaded or low-flow microhabitats, fragments must contend with competitors that thrive on the sediment and dead coral skeletons. Those, for instance, include turf algae (*56*), which are the dominant competitors for space (*67*). Over recent decades, human-driven stressors have shifted this balance in favor of algae, leading to widespread coral decline (*68*). The ability to erect protective skeletal walls provides corals with a crucial survival strategy under adverse conditions, allowing juvenile colonies or fragments to secure critical space before resuming normal expansion. These defensive walls, however, are inherently thin and fragile. If overrun by competing organisms, the protective matrix gradually deteriorates and is lost. Conversely, if the coral prevails, the defensive structures are either overlaid or remodeled into the regular growth pattern. It is noteworthy that new skeletogenesis in the defensive growth part does not follow the preexisting pattern but instead creates a new one, which later can be remodeled.

The defensive walls that we described here are devoid of photosynthetic symbionts, which support an ecologically tuned program: deploying metabolically independent structural shields at the expense of immediate symbiont productivity. By contrast, lateral expansion through recurrent fusing microparticles reflects a conservative stitching mechanism optimized for steady, incremental area gain, whereas vertical growth, requiring coordinated polyp initiation in a 3D environment, depends on additional developmental programs involving 3D logic of morphogenesis.

The phenomenon of defensive growth is markedly different from colony rejuvenescence, which occurs when some coral territories containing polyps die and are later reconquered by the live tissue as previously reported: The abandoned corallites tend to be reused by freshly emerging polyps (*69*). This is exactly what we also observe in our coral colony regeneration experiments, where nearly half of the colony becomes reestablished after local death, and the underlying dead skeleton is reused and readapted for new live polyps.

In vertical growth, the fusion of discrete skeletal microparticles [conceptually related to previously observed spherulites (*16*, *57*) and microdumbbells (*6*)] is complemented by more homogeneous accretion, demonstrating that coral skeletogenesis relies on a composite strategy that integrates modular particle assembly with continuous matrix deposition. The observed skeletal microparticles initially form RADs, which expand and become further polished, integrated, and smoothened by layered matrix deposition via TDs. Thus, our results confirm modular and alternating assembly rather than continuous bulk mineralization, lending support to theories of biomineralization, which necessarily include a step of skeletal particle generation and fusion (*63*, *64*).

Our findings of microscale growth dynamics are in line with a set of previous works that have investigated the skeletal development of newly settled primary polyps, focusing on the earliest stages after larval settlement (*6*, *48*, *49*). Being tiny and relatively transparent, just about 2 to 3 mm in diameter, these initial skeletal structures were studied using optical microscopy, complemented by the incorporation of fluorescent dyes (*70*), SEM, and end-point microCT (*6*, *51*, *63*). These studies revealed that the very first skeleton of a single primary polyp forms from numerous discrete skeletal particles, which are subsequently fused together and overlaid by matrix layers that accrete over time. These observations were also important and instrumental in building causal links between ocean conditions and skeletal programs by introducing controlled changes in pH and carbonate chemistry during nascent polyp growth (*50*, *51*).

On a different note, our microCT time series demonstrate that corals reallocate mineral resources in space and time: Deposition can be transiently redirected during defensive responses or optimum-searching regular expansion. This oscillating “exploratory colony edge” behavior resembles the axon growth cone (*71*), or oscillating yet directional movements of amoeboid cells searching for the optimal path (*72*, *73*). By analogy, the coral colony is actively exploring the most optimal direction of growth, which might change over time. Such “searching” retraction and regrowth of coral colonies is accompanied by partial uptake of already allocated mineral matrix, indicating that corals actively manage skeletal material at the colony scale in response to measured risk, damage, and threat. Critically, these transient reversals and reallocations would remain invisible in static imaging approaches; only longitudinal live microCT enables the temporal resolution necessary to detect, quantify, and interpret the oscillatory nature of this growth strategy. Understanding in the future how energetic cost, symbiont function, and local habitat chemistry constrain these allocation decisions will be essential to link skeletal dynamics to fitness and resilience. Plasticity in skeletal strategy may underpin differential resilience among species and morphologies, partly explaining the variety of nascent coral architectures (*74*).

Last, the principles that we document may generalize to other biomineralizing systems: Modular assembly, rhythmic secretion, and resource reallocation are strategies that could be conserved across phyla (*75*). While our study focused on reef-building corals, the potential of longitudinal microCT extends broadly to other mineralizing organisms. In mollusks, for example, repetitive scans could track shell accretion dynamics and capture how growth lines emerge and remodel under fluctuating environmental conditions (*76*). Colonial bryozoans present another promising system (*77*), where microCT could visualize the expansion of zooids and the assembly of skeletal modules in three dimensions, offering parallels to coral polyp-driven growth. Similarly, echinoderms, serpulid polychaetes, and other sessile calcifiers undergo cycles of deposition and repair that are now understood only from end-point analyses (*78*–*80*). Applying nondestructive, longitudinal imaging to these systems would allow direct comparisons of how mineralization strategies vary across lineages and may uncover shared principles of modular construction, repair, and plasticity. In this way, longitudinal microCT offers a powerful comparative framework for probing the universal logic of biomineralization across diverse taxa.

While live microCT offers suitable resolution and continuity, it is not without limitations: Imaging intervals must balance temporal resolution with potential stress from repeated scanning. Corresponding tests and the identification of the optimal scanning regime should be performed before experiments, as was done in our approach, or when establishing any longitudinal experiments involving potentially harmful exposure of live specimens (*81*, *82*). To further approach the molecular and cellular mechanism, live microCT should be combined with complementary modalities. Correlative imaging that links longitudinal microCT with fluorescent calcium tracers (*83*), higher-resolution electron microscopy (*84*, *85*), or simultaneous imaging of metabolic dynamics would clarify whether colony redistribution of microelements reflects vesicle-mediated secretion, skeletal particle attachment, or other processes. In the future, integrating single-cell (*52*, *86*) or spatial transcriptomics of the calicoblastic layer before and after induced defensive responses could reveal regulatory networks and ion transporters that execute these programs.

## MATERIALS AND METHODS

### Coral samples and cultivation conditions

*S. pistillata* and *P. damicornis* were obtained from Haus des Meeres, a public aquarium (Vienna, Austria). The original coral colonies originate from Bleijdorp Zoo, Rotterdam, The Netherlands, as of 2012. Because our aim was to repeatedly image the same individual colonies and capture oscillatory dynamics in skeletal microstructure, we selected species that are both experimentally tractable and robust in aquaculture. *S. pistillata* and *P. damicornis* fulfilled these criteria: They are readily maintained under controlled conditions, phylogenetically related, and yet biologically distinct. This combination enabled us to generalize shared growth principles while also identifying species-specific strategies of skeletal deposition.

Physical and inorganic chemical parameters of the culture water were analyzed weekly and stayed with ranges suitable for cultivation photosynthetic scleractinian corals. Nitrate, <20 mg/liter; phosphate, <0.1 mg/liter; salinity, 33 practical salinity units; temperature, 25°C; light, 100 μM/m^2^ per second (photosynthetically active radiation); and current, 2 to 5 cm/s.

### Production of nubbins

To produce the nubbins, the mother colony was removed from the aquarium and placed on a work desk in a dish filled with aquarium water. Colonies were fragmented in air with side-cutting pliers into nubbins of ~5 to 7 mm in diameter and consisting of fewer than 50 polyps, aiming for uniform size to improve experimental replication. The pliers were washed with freshwater and sterilized immediately before use and were free of mineral oil. Plastic attachment bases for the coral nubbins were prepared in advance from polyethylene terephthalate (PET) sheets of 0.5-mm thickness. PET was chosen as it is not buoyant in seawater and can be glued effectively.

Freshly cut nubbins were placed on laboratory tissue with the attachment side facing downward to absorb excess water and left for 1 min. During this time, drops of gel-type cyanoacrylate glue were applied to the clean and dry PET strips. Nubbins were grasped with dry plastic forceps and placed on the glue bed so that their bases were fully covered. After 1 min, seawater was gently dripped onto the nubbins using a pipette to accelerate curing, and dripping was repeated every minute to prevent drying of polyps until the glue solidified. Fixing positions in the water tank were prepared in advance to orient the nubbins toward the light source and expose them to water current.

To study defensive strategies of corals, a group of nubbins was placed in a separate tank where algal overgrowth was observed. Algae rapidly colonize free substrates and compete with corals for space. All other cultivation parameters were maintained as in the main culture system, including seawater chemical composition, light intensity, and water flow.

### Preparation of nubbins for microCT experiments

Nubbins grown on plastic strips could be mounted on experimental equipment. These small colonies expanded rapidly, reaching ~1 cm within a few weeks, accompanied by an increase in polyp number, the formation of new skeletal chambers, and the expansion of preexisting chambers. This growth system was therefore suitable for longitudinal study.

Coral nubbins were transported in containers filled with seawater, which allowed repeated measurements of the same individuals. For scanning, the PET plastic film with attached nubbins was cut to isolate each specimen. Each nubbin, attached to PET film, was glued onto a plastic lid that was subsequently screwed onto a plastic 5-ml tube filled with sand to prevent floating in water. This tube was placed inside a 50-ml Falcon tube of larger diameter.

Before scanning, the lid was unscrewed from its 5-ml tube holder and mounted inside the 50-ml Falcon tube filled with seawater, ensuring stability of the coral in a natural aqueous environment during scanning while permitting rotation near the x-ray source. Immediately after each CT scan, microphotographs of the scanned nubbin were taken using a stereomicroscope (Levenhuk DTX RC3), enabling simultaneous tracking of polyp formation and skeletal growth. Top-view microphotographs were processed using focus-stacking to keep both the top and bottom parts of the nubbin in focus on the final photo.

For assessing the influence of time scan and scan frequency, three groups of four to five nubbins for each condition were used. Horizontal and vertical growth as well as microlevel growth patterns were observed in all groups consistently: *P. damicornis*, 10 nubbins; and *S. pistillata*, 24 nubbins. For defensive growth experiment the group of *S. pistillata*, four nubbins were used. All these nubbins were scanned multiple times with intervals ranging from 1 week to 2 months. Therefore, hundreds of scans were generated during longitudinal courses focusing on tracking individual nubbins over time. Many more nubbins were observed visually and appeared consistent with the scanned groups. To control the influence of environmental factors and scans, each group was accompanied by control nonscanned nubbins growing in the same water tanks under the same cultivating conditions.

### X-ray microCT

The microCT measurement was done in the Laboratory of x-ray microCT and nano–computed tomography (nanoCT; CTlab) at Central European Institute of Technology, Brno University of Technology (CEITEC BUT). The device used for this experiment is GE v|tome|x L240 equipped with a 180-kV/15-W maximum power nanofocus x-ray tube and high contrast flat panel detector (dynamic 41|100 with 4000 × 4000 pixels, each 100 μm by 100 μm in size). The geometry of the experiment was designed with a voxel resolution 6 μm for both species to have the whole nubbin in the field of view and, at the same time, to be able to track small changes. The resolution was the same during all longitudinal scans. Before every measurement, the calibration with ruby ball phantom was performed to be able to align data properly. The sample-source distance was optimized considering the radiation damage and noise in CT data. The energy of the x-ray tube aimed for high voltage and low current to minimize the radiation damage to the living tissue: 140-kV acceleration voltage and 100-μA tube current. The x-ray spectrum was filtered by 0.2-mm copper filter to avoid low-energy photons, because of beam-hardening artifacts and radiation damage simultaneously. The exposure time was 334 ms, and the number of projections varied with the resulting scanning time from 2 to 15 min (Table 1).

**Table 1.** Number of projections acquired under scanning regimes with varying scan durations.

Scanning time, min	Number of projections
2	359
5	898
6	1000
10	1796
15	2694

Tomographic reconstructions were performed using GE phoenix datos|x 2.0 3D computed tomography software, which uses a filtered back-projection algorithm as the core reconstruction method. The reconstructed volumes were subsequently analyzed in VGStudio MAX 2024.3 (Volume Graphics, Germany) for data segmentation, alignment, and quantitative analysis.

In the first step, different noise-filtering approaches were evaluated as all scans were acquired with relatively short acquisition times (2 to 15 min) to minimize radiation damage. Four filters—Gaussian, convolutional, median, and bilateral—were tested. Their performance was evaluated by comparing signal-to-noise ratio (SNR), contrast-to-noise ratio (CNR), and image sharpness.

In subsequent steps, combinations of these filters were also compared. The optimal balance between SNR, CNR, and sharpness was achieved using a combination of median and bilateral filtering. However, this procedure proved time-consuming as more than 280 scans were analyzed over the course of 1 year.

Therefore, final segmentation was performed using the “Surface Determination” module in VGStudio MAX, based on histogram thresholding of the CT data. The filtered datasets were used as references to determine appropriate histogram thresholds. After generating the 3D models of the nubbins, further analyses included nominal/actual comparisons to visualize growth increments between consecutive scans and displacement analysis to track tissue retraction and expansion.

### Staining of soft tissues

For the visualization of soft tissue, the nubbins were fixed in 4% formaldehyde in phosphate-buffered saline overnight. Subsequently, samples were dehydrated in incrementally increasing ethanol concentrations (30, 50, and 70%), 2 hours in each concentration. Then, nubbins were transferred, into 1% PTA in 90% methanol for tissue contrasting. The PTA-methanol solution was changed every 2 to 3 days for 10 days. The nubbins were then put in 70% ethanol and scanned in the ethanol. Scanning of stained nubbins was performed using the same machine as for live nubbins, but with the different scanning parameters: The energy of the x-ray tube was 70 kV, 280 μA. The exposure time was 334 ms, and three projections were captured in one position to reduce the noise in the data. Two thousand images were captured over 360°. The voxel resolution was 5 μm.

### X-ray nanoCT

To confirm coral skeleton dynamics, we scanned the skeleton of the dead *P. damicornis* nubbin, previously imaged in longitudinal microCT experiment. We cut off a piece of the nubbin 2.5 mm by 2.5 mm that was glued to the aluminum rod for the nanoCT scan. NanoCT scan was performed using Rigaku Nano3DX machine using rotational Molybdenum target (50 kV, 24 mA). The exposure time was 5 s, and 800 images were taken over 180°. The isotropic voxel size of the reconstructed dataset was 1.47 μm.
