# Supplementary material for: Coral growth, retraction, defense, and regenerative strategies revealed by live microCT
Source: Sci Adv. 2026 May 27;12(22):eaee3183. doi: 10.1126/sciadv.aee3183 (PMC13215181; doi:10.1126/sciadv.aee3183)
Supplement: Supplementary file 1 — Figs. S1 to S4 Legend for movie S1 [file sciadv.aee3183_sm.pdf]

Science Advances
AAAS

Supplementary Materials for

Coral growth, retraction, defense, and regenerative strategies revealed by live microCT

Karina Araslanova *et al.*

Corresponding author: Igor Adameyko, igor.adameyko@meduniwien.ac.at;
Jozef Kaiser, jozef.kaiser@ceitec.vutbr.cz

Sci. Adv. **12**, eaee3183 (2026)
DOI: 10.1126/sciadv.aee3183

The PDF file includes:

Figs. S1 to S4
Legend for movie S1

Other Supplementary Material for this manuscript includes the following:

Movie S1

Growth dynamics of individual *Stylophora pistillata* nubbins under varying μCT scanning regimes

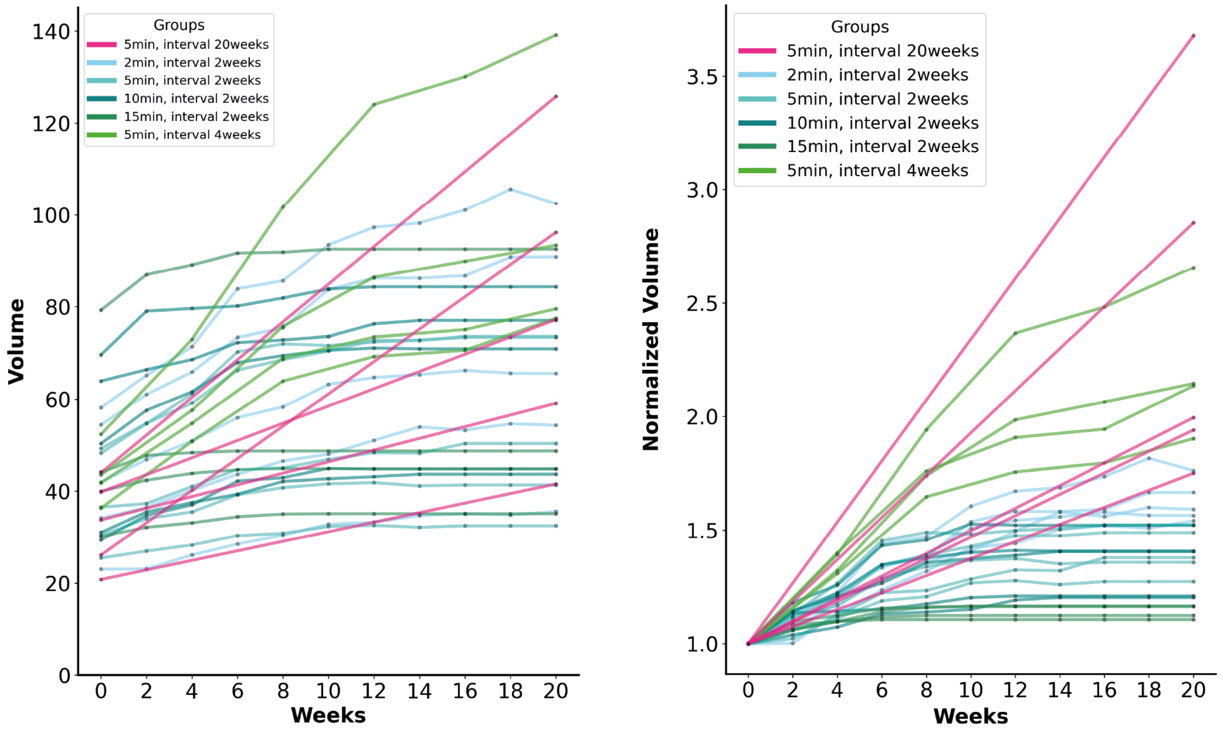

Fig. S1.

Longitudinal analysis of skeletal growth of *Stylophora pistillata* nubbins under varying microCT scanning regimes. Different colored lines represent groups exposed to different scanning intervals (as indicated in the legend). Volume represented by growth trajectories (left) and final normalized skeletal volume (right) of coral nubbins scanned under 6 regimes with different scan duration and scan frequency.

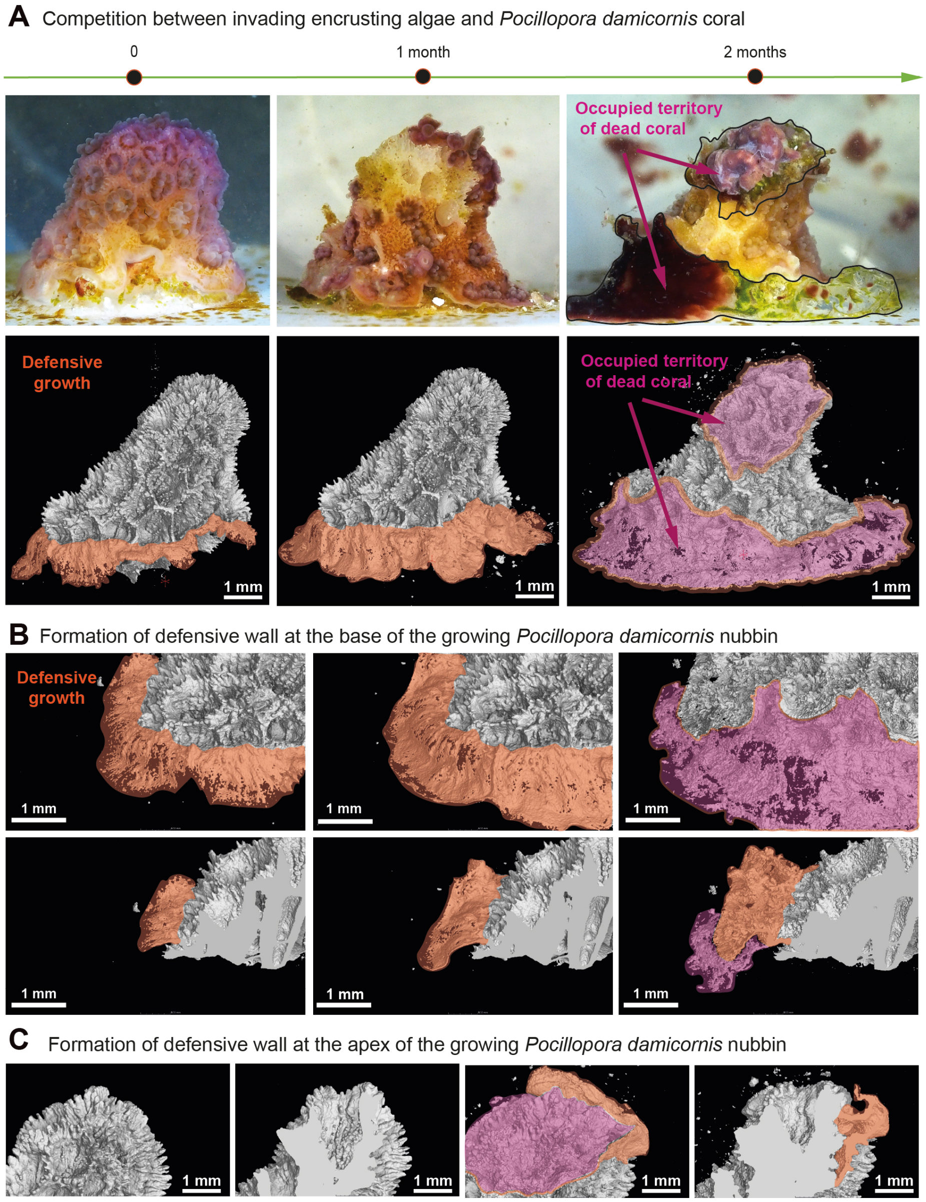

Fig. S2.

Defensive morphological responses of *Pocillopora damicornis* to competition with encroaching algae. Related to Fig. 2

(**A**) Photograph and microCT time-series (0-2 months) of an interaction showing encrusting algae invading and occupying the skeleton of a *Pocillopora damicornis* nubbin, which responds with defensive growth at the interface. (**B**) Sequence of defensive wall formation at the base of a nubbin. The coral constructs a barrier to isolate the algal-occupied territory; when the algae overgrow this barrier, a subsequent wall is formed. MicroCT reconstruction with clipping plane on the 3D model showing internal area. (**C**) The same defensive process of sequential barrier formation occurs when the interaction takes place at the top of the nubbin.

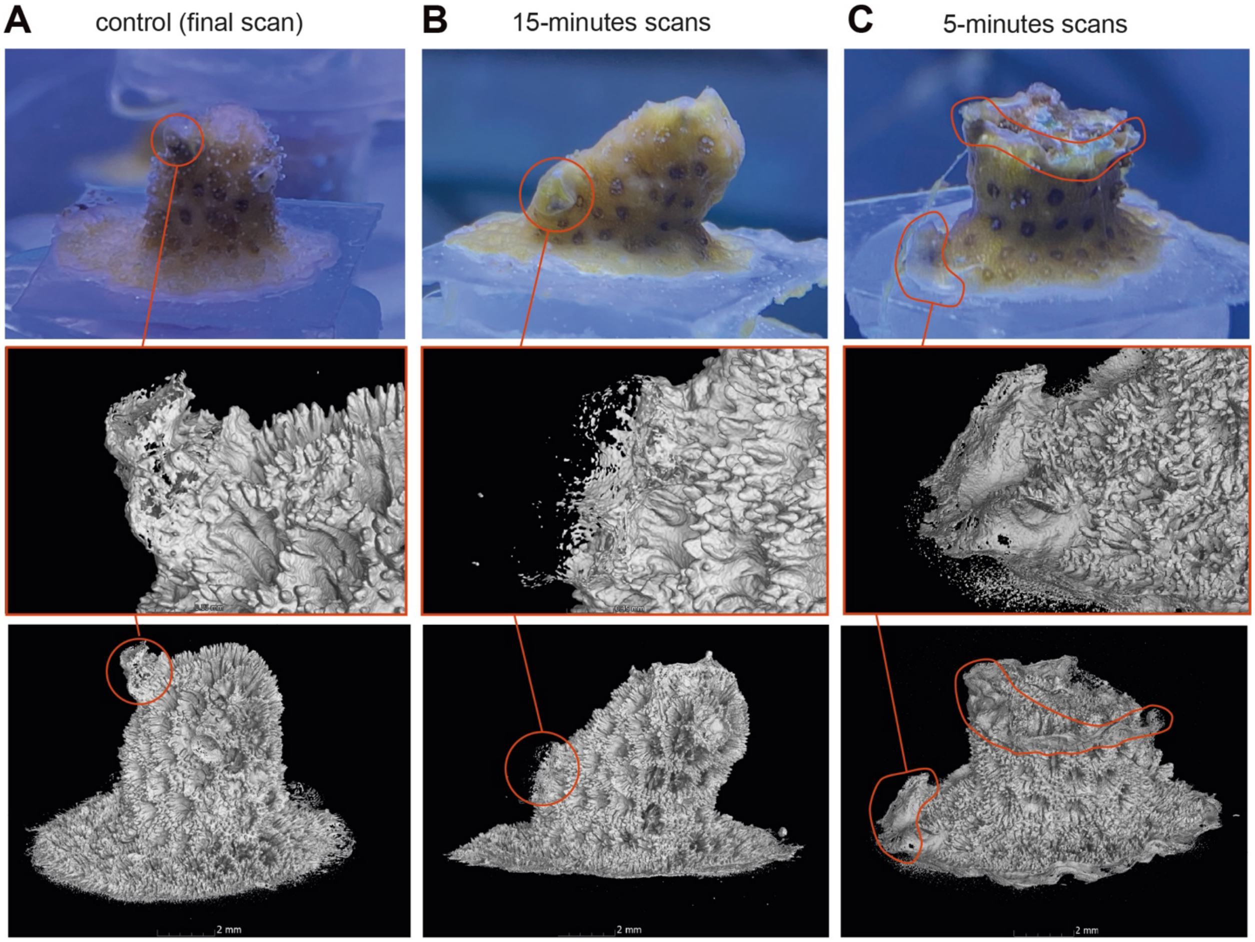

Fig. S3.
Defensive growth appears independently of scanning frequency or duration. Related to Fig. 2.
(**A**) Control nubbin scanned only once at the endpoint of the experiment. Note the formation of defensive structures (in red circle). (**B**) Nubbins scanned 5 times for 15 minutes. (**C**) Nubbins scanned 5 times for 5 minutes.

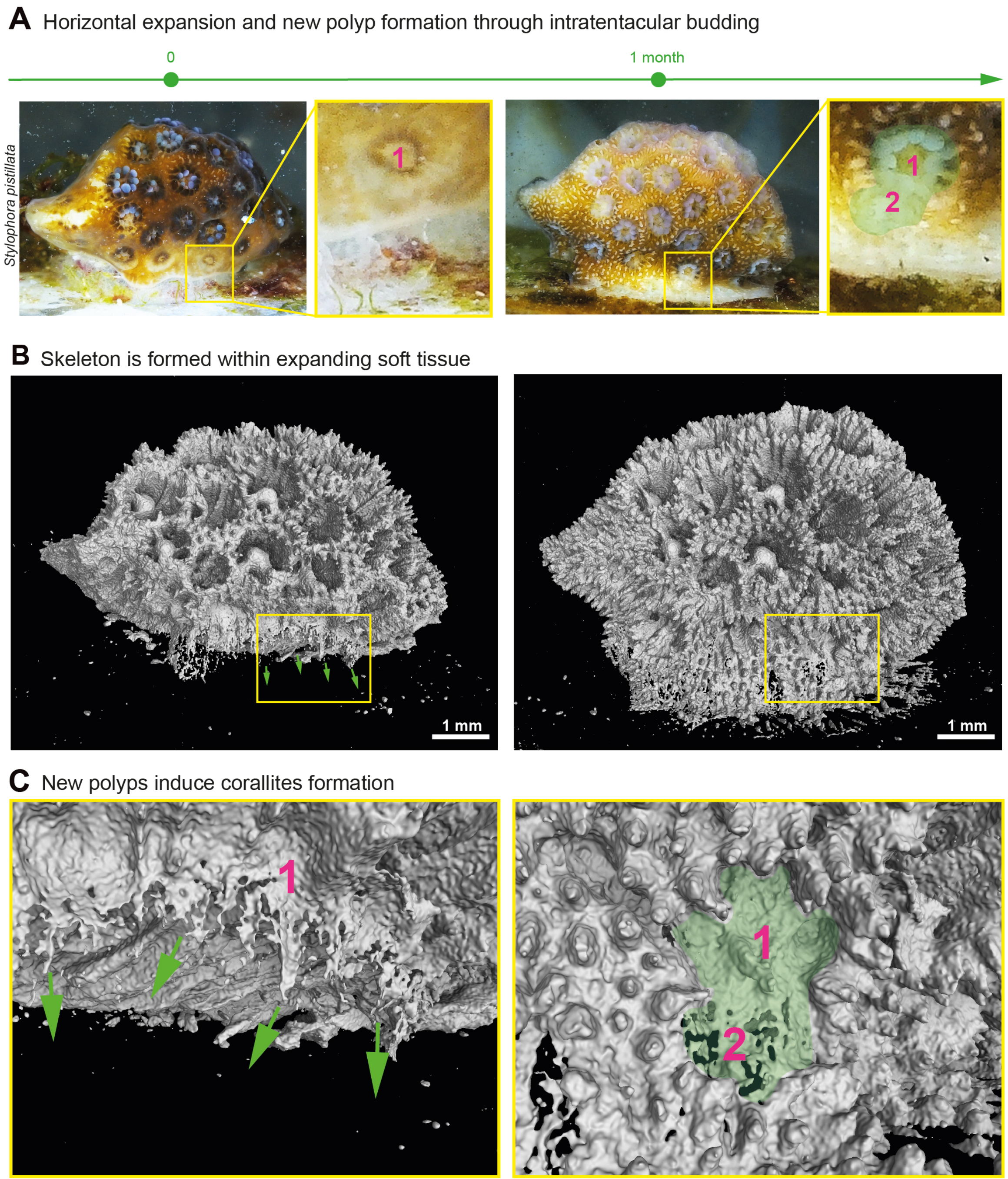

Fig. S4.

Horizontal expansion of *Stylophora pistillata* nubbin. Related to Fig. 3.

(**A**) Soft tissue spreads and initiates new polyp formation at the expanding margin. (**B**) The expanding tissue deposits skeleton directionally (green arrows) and conquers the substrate surface. Note the high complexity of the skeletal microstructures forming at the surface of expanding skeleton (similar to *Pocillopora damicornis* in Fig. 3). (**C**) Magnified view showing the formation of the new polyp at the expanding margin.

Movie S1. (separate file) 3D reconstructions capturing 5 months of *Pocillopora damicornis* nubbin growth.
